# Tyrosine Kinase Inhibitors in Cancer: Breakthrough and Challenges of Targeted Therapy

**DOI:** 10.3390/cancers12030731

**Published:** 2020-03-20

**Authors:** Charles Pottier, Margaux Fresnais, Marie Gilon, Guy Jérusalem, Rémi Longuespée, Nor Eddine Sounni

**Affiliations:** 1 Laboratory of Tumor and Development Biology, GIGA-Cancer and GIGA-I3, GIGA-Research, University Hospital of Liège, 4000 Liège, Belgium; marie.gilon@student.uliege.be (M.G.); nesounni@uliege.be (N.E.S.); 2 Department of Medical Oncology, University Hospital of Liège, 4000 Liège, Belgium; G.Jerusalem@chu.ulg.ac.be; 3 Department of Clinical Pharmacology and Pharmacoepidemiology, University Hospital of Heidelberg, 69120 Heidelberg, Germany; Margaux.Fresnais@med.uni-heidelberg.de (M.F.); remi.longuespee@med.uni-heidelberg.com (R.L.); 4 German Cancer Consortium (DKTK)-German Cancer Research Center (DKFZ), 69120 Heidelberg, Germany

**Keywords:** cancer, oncology, pharmacology, tyrosine kinase inhibitors

## Abstract

Receptor tyrosine kinases (RTKs) are key regulatory signaling proteins governing cancer cell growth and metastasis. During the last two decades, several molecules targeting RTKs were used in oncology as a first or second line therapy in different types of cancer. However, their effectiveness is limited by the appearance of resistance or adverse effects. In this review, we summarize the main features of RTKs and their inhibitors (RTKIs), their current use in oncology, and mechanisms of resistance. We also describe the technological advances of artificial intelligence, chemoproteomics, and microfluidics in elaborating powerful strategies that could be used in providing more efficient and selective small molecules inhibitors of RTKs. Finally, we discuss the interest of therapeutic combination of different RTKIs or with other molecules for personalized treatments, and the challenge for effective combination with less toxic and off-target effects.

## 1. Introduction

### 1.1. Description of Receptor Tyrosine Kinases and Downstream Signaling Pathways 

**Figure 1:**
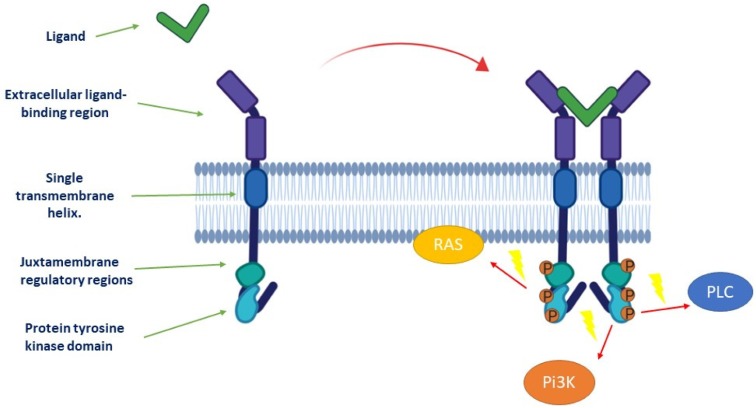
Activation of tyrosine kinase receptor. Ligand binding stabilizes connections between monomeric receptors to form an active dimer, which in turn activates the intracellular kinase. Three main effectors can be activated later: phosphoinositide 3-kinase (Pi3K), rat sarcoma (RAS), and phospholipase C (PLC).

Receptor tyrosine kinases (RTKs) are key regulators of cellular processes and their role in the pathophysiology of many diseases is well recognized. The known human RTKs are classified into twenty subfamilies and have a similar molecular architecture (Figure 1), with an extracellular ligand-binding region and a single transmembrane helix. The cytoplasmic region contains the protein tyrosine kinase (TK) domain with additional carboxy-(C-)terminal and juxtamembrane regulatory regions. 

Activation of RTK (Figure 1) involves ligand binding to stabilize connections between monomeric or oligomeric receptors and form active dimers or oligomers, which in turn activate the intracellular kinase. RTK activities are mainly dependent on signaling molecule phosphorylation and activation of transcription factors that mediate target gene expression in response to ligands [1]. These activities can also be regulated by receptor internalization and recycling during physiological and pathological processes. 

**Figure 2:**
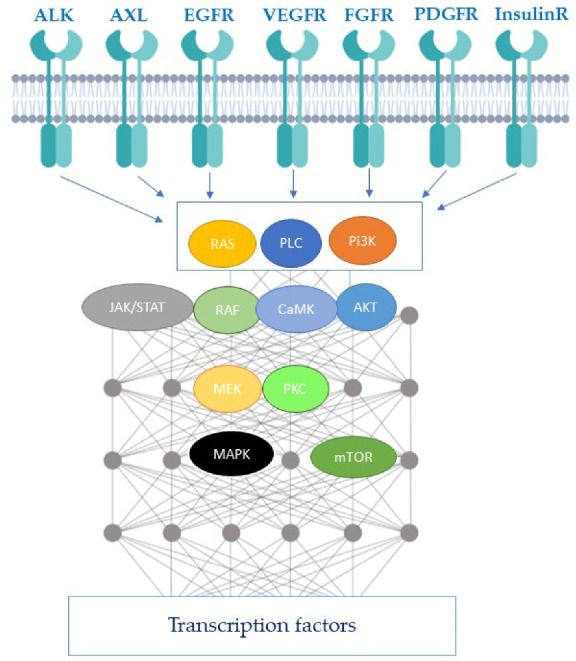
Receptor tyrosine kinase (RTK) network modeling proposed by the Kitano’s “bow tie”. The set of RTKs (input layer) influences a small number of intermediaries, such as mitogen-activated protein kinases (MAPK), phosphoinositide 3-kinase (Pi3K), and Ca^2+^ signaling (core processes), which leads to the activation of a complex signaling network implicating Pi3K/protein kinase B (AKT)/mechanistic target of rapamycin (mTOR), rat sarcoma (RAS)/MAPK, Janus kinase (JAK)/STAT, and phospholipase C (PLC)/Ca^2+^/calmodulin-dependent protein kinase-protein kinase C (CaMK-PKC) pathways with their numerous crosstalks. The result of the signaling cascade leads to a transcriptional control.

The signaling pathways of the RTKs are complexes and involve an increasing number of biochemical reactions and molecular mediators in complex signaling networks. For instance, epidermal growth factor receptor (EGFR) signaling network involves 211-biochemical-reactions and 322 signaling molecules [2]. In fact, attempts of modeling of this network seem to be very complex due to the requirement of more sophisticated spatial and stochastic aspects. An example of RTK network modeling was proposed by Kitano’s “bow tie” or “hourglass” (Figure 2) [1]. In this model, the set of RTKs (input layer) influences a small number of intermediaries, such as mitogen-activated protein kinases (MAPK), phosphoinositide 3-kinase (Pi3K), and Ca^2+^ signaling (core processes). RTKs mainly activate the Pi3K/protein kinase B (AKT)/mechanistic target of rapamycin (mTOR), rat sarcoma (RAS)/MAPK, Janus kinase (JAK)/signal transducer and activator of transcription protein family (STAT), and phospholipase C (PLC)/Ca^2+^/calmodulin-dependent protein kinase-protein kinase C (CaMK-PKC) pathways, and downstream effectors of multicellular processes during cancer progression [3]. Pi3K/AKT/mTOR pathway controls cell growth, metabolism, and survival, and is essential for maintaining pluripotency. The RAS/MAPK pathway is a central regulator of metabolism, cell cycle, cell proliferation, differentiation, and migration. STAT is known to control the signaling activated by lymphokines, the platelet-derived growth factor (PDGF), the epidermal growth factor (EGF), or the fibroblast growth factor (FGF), and is therefore involved in many cellular changes Finally, PKC is also involved in the regulation of survival, proliferation and cell motility [3,4,5]. It is important to note the complex crosstalks between these pathways. For example, the Pi3K/AKT and RAS/MAPK pathways interfere in various nodes including extracellular signal-regulated kinases (ERK), and regulate themselves through a positive and negative feedback, depending on the cellular context. These two pathways can also be activated by molecules of the STAT pathway, and finally, PKC has been shown able to activate MAPK. Many neoplastic characteristics (proliferation, metabolic anomaly, migration, etc.) are therefore regulated at least in part by the intracellular signaling triggered by RTKs, and some key kinases of these pathways (Pi3K, RAS, JAK, STAT, etc.) are frequently mutated in cancers [6,7,8]. Since molecular deregulation of cell signaling is essential for the acquisition of cancer hallmarks, classifying tumors according to these anomalies is complementary to histological classification. This molecular classification is becoming more and more important. Thus, in certain clinical trials known as “agnostics”, the therapeutic strategy no longer depends on the histology, but only on the molecular anomalies. These clinical trials are currently investigating inhibitors of BRAF, human epidermal growth factor receptor-2 (HER2), and Pi3K/AKT/mTOR or rapidly accelerated fibrosarcoma (RAF)/mitogen-activated protein kinase kinase (MEK) pathways on mutated tumors [9].

### 1.2. Classification of Receptor Tyrosine Kinase Inhibitors

Cancer cell proliferation has been proposed to follow the Darwinian selection in order to continue proliferation in harsh conditions and changes imposed by tumor microenvironment (TME) [10]. Drug targeting intracellular signaling have been developed to target the hubs of these molecular pathways. Among these drugs, receptor tyrosine kinase inhibitors (RTKIs) are a large family within these targeted drugs and have been used clinically with numerous successes since 2001. They generally target the active site of the kinase and thereby prevent the phosphorylation of intracellular targets, which are often involved in cell proliferation or angiogenesis [11,12]. As of August 2019, 43 RTK inhibitors were approved by the Food and Drug Administration (FDA) for oncological indications (Table 1) [13]. Reversible inhibitors are usually distinguished from irreversible ones, which bind covalently with or near an adenosine triphosphate (ATP) binding site. Among the non-covalent inhibitors, the majority are ATP-competitive inhibitors that link to active conformations (type-I inhibitors). ATP binding sites are generally conserved, therefore selectivity can be achieved by targeting poorly preserved residues, particularly residues flanking the hinge. Type-II inhibitors bind to a site adjacent to the ATP site of inactive kinases and maintain their inactive conformation. This type of inhibitor is usually nonselective. Allosteric inhibitors (type III) inhibit kinases by binding to an allosteric site, remote from the ATP site and the hinge, and are highly selective [14]. New substrate-directed inhibitors or type-IV RTKIs, which target substrate-binding site in a reversible manner, are under development. Finally, covalent kinase inhibitors bound irreversibly with the kinase active site, also called type-V inhibitors, and have the advantage to be potent and have reduced off-target side effects [15].

This review discusses the current challenges of the use of RTKIs in clinic and the potential use of new emerging technological advances for the design of efficient RTKIs therapies. 

## 2. Current Place of Receptor Tyrosine Kinase Inhibitors in Oncological Treatments

RTKIs occupy an important place in precision oncology, even if their effectiveness is limited by the acquisition of resistance mechanisms. This section describes the advent of RTKIs in non-small cell lung cancer (NSCLC) and other types of cancer, and the mechanisms of resistance.

### 2.1. Evolution of Receptor Tyrosine Kinase Inhibitor Use in Non-Small Cell Lung Cancer

The use of RTKIs in NSCLC was reported in clinical trials with the first generation, EGFR reversible inhibitors of ATP-binding sites (gefitinib and erlotinib in cancer patients). Compared with chemotherapy, these treatments improved patient survival by 50% (overall survival (OS) of 30.5 months versus 23.6 months) [20]. The second generation of RTKIs is composed of irreversible inhibitors (afatinib and dacomitinib) with greater affinity for the EGFR kinase domain, which also inhibits other members of the HER family to which the EGFR belongs. Afatinib doubles survival compared to chemotherapy (median progression-free survival (mPFS) of 11.1 months versus 6.9 months) [21]. Clinical studies showed longer survival with dacomitinib than with gefitinib (mPFS of 14.7 months versus 9.2 months and median OS (mOS) of 34.1  months versus 26.8  months, respectively) [20,22]. Side effects, including skin irritation or ulceration, and gastrointestinal toxicity (diarrhea, constipation, severe nausea, and vomiting) are more frequently severe with afatinib and dacomitinib.

The most common mechanism of resistance to these drugs is a secondary mutation of EGFR kinase domain. Therefore, a third generation EGFR-RTKI, osimertinib, was designed to have more affinity for mutated receptors. Compared to first generation EGFR RTKI, osimertinib significantly improved PFS (18.9 months versus 10.2 months), with a favorable tolerability profile [20,23,24].

The current recommendation of the European Society for Medical Oncology (ESMO) for NSCLCs with an EGFR-activating mutation is the treatment with erlotinib, gefitinib, and afatinib or osimertinib as first-line therapy [25]. Combination of gefinitib with chemotherapy (carboplatin/pemetrexed) is also recommended in the first line therapy for NSCLC. Other options are under investigation but not recommended yet, such as the combination of erlotinib with bevacizumab or ramucirumab (anti-angiogenic antibodies targeting the vascular endothelial growth factor receptor (VEGFR)). Indeed, the drug combination targeting EGFR and VEGFR appears to be synergistic, probably because of the crosstalk between their signaling pathways and vascular normalization induced by anti-angiogenic therapy, which can in turn increase intra-tumor concentration of RTKI [26]. After systemic progression, the recommended second-line treatment is osimertinib in the case of a T790M mutation in exon 20 [25]. In the absence of this mutation, the guidelines recommend chemotherapy (platinum-based) with or without bevacizumab, or immunotherapy (atezolizumab) [25].

Many early phase clinical studies are investigating new combination therapies. For example, some ongoing clinical trials are investigating the combination of a first generation EGFR RTKI (gefitinib) with osimertinib as a first line treatment [27]. In addition, chidamide, an histone deacetylase (HDAC) inhibitor, is tested in combination with several RTKIs of EGFR, after promising pre-clinical results showing a synergistic action (chidamide notably prevents the activation of pathways such as RAS/MAPK and Pi3K/AKT) [28]. Further investigations are evaluating the efficacy of new third generation EGFR RTKIs (nazartinib, rociletinib, avitinib), non-selective RTKIs targeting several RTKs, including EGF and VEGF (sorafenib, anlotinib, momelotinib), or RTKIs which target the c-MET hepatocyte growth factor (capmatinib, tepotinib), the AXL receptor tyrosine kinase (gilteritinib), and Janus kinase 2 (JAK2) (pacritinib, momelotinib). While positive results are expected from theses ongoing studies, it should be noted that in the recent years, most strategies combining RTKIs have failed in clinical phases for lack of efficiency or tolerability [29].

### 2.2. Current Indications of Receptor Tyrosine Kinase Inhibitors in Other Types of Cancer

NSCLC with mutated EGFR is an example that can summarize the evolution of RTKIs that showed limited efficacy during the last twenty years. The situation is the same for other tumors, with the exception of chronic myeloid leukemia, which can be “cured” with one or more RTKIs. 

For renal cell carcinoma (RCC), anti-angiogenic RTKIs, such as sunitinib or pazopanib, are recommended by the ESMO as first-line therapy in patients with favorable prognosis. In case of relapse after treatment with RTKI, the second line treatment is either immunotherapy (nivolumab) or another RTKI (cabozantinib or axitinib) [30]. Effectiveness of the second line is explained by dual inhibitory effects on VEGFR2, and on MET or MAPK, which are activated in cancer cells resistant to first line RTKIs [31,32].

In metastatic HER+ breast cancer, lapatinib can be used as a first line in addition to trastuzumab in selected patients due to the high toxicity of this treatment. In the second line therapy, lapatinib seems less effective than the antibody-dug conjugate trastuzumab emtansine, T-DM1 [33].

Systemic therapy for hepatocellular carcinoma relies mainly on multitarget kinase inhibitors. In the first line treatment, sorafenib (VEGFRi, PDGFRi) or lenvatinib (targeting VEGFRi, FGFRi, PDGFRi) are recommended, whereas cabozantinib (RETi, growth arrest-specific 6 (GAS6)i), regorafenib (VEGFRi, FGFRi and PDGFRi), or immunotherapy were used in second line [34]. 

In metastatic colorectal cancer, regorafenib, a multitarget kinase inhibitor (EGFRi, PDGFRi, FGFRi), is the first RTKI that demonstrated a modest benefit in survival (6.4 versus 5 months) in the third line after failure of antibodies targeting EGFR and VEGFR and chemotherapy [35]. Imatinib (breakpoint cluster region (BCR)-Abelson (ABL) inhibitor) revolutionized the prognosis of gastrointestinal stromal tumors expressing the RTK, KIT (CD117), with a mOS longer than 5 years [36], and is the current first line therapy. After relapse, sunitinib and regorafenib can be used [37]. 

Lenvatinib (VEGFRi) and sorafenib are considered as first line therapy for differentiated thyroid cancers resistant to radioactive therapy with a significant improvement of PFS (10.8 months with sorafenib versus 5.8 months with placebo, mPFS 18.3 months with lenvatinib versus 3.6 months with placebo) [38,39]. For progressive metastatic medullar thyroid carcinoma, cabozantinib and vandetanib (EGFRi, RETi) are the first-line systemic treatments (PFS are 30.5 months with vandetanib versus 19.3 months with placebo, and 11.2 months with cabozantinib versus 4.0 months with placebo) [40].

First line standard treatment for unresectable stage III/IV BRAF V600 mutated melanoma is BRAF RTKI (vemurafenib, dabrafenib, encorafenib) combined with MEK RTKI (cobimetinib, trametinib, binimetinib) with an mPFS of the order of one year. Second line treatment is based on immunotherapy alone [41].

To conclude, the best achievement of RTKIs in cancer treatment concerns chronic myeloid leukemia (CML), since the survival rate of patients treated with imatinib at 10 years is 83.3% [42]. There are some evolutionary explanations to CML exception. Cell population of CML is homogenous and is therefore not subjected to an allopatric speciation process. One dominant oncogene is present throughout the population, and clonal changes are progressive and orderly during the chronic phase of the disease. These elements explain why the CML evolves in a clonal and stepwise manner, in contrast to the dynamic and stochastic evolution of solid tumors [43]. Pre-clinical studies have identified other causes of resistance, such as compensatory hyperactivation of anti-apoptotic pathways (e.g., CRS), the involvement of efflux proteins (e.g., P-glycoprotein or the human organic cation transporter 1 (hOCT1)) [43]. In the case of low-risk chronic CML, first generation RTKIs (imatinib) or second generation RTKIs (nilotinib, dasatinib and bosutinib) are recommended, whereas only second generation RTKIs are indicated for CML in chronic phase with high risk, and panotinib and other third-generation RTKIs are reserved for the second line of treatment. [44].

### 2.3. Mechanisms of Resistance to Receptor Tyrosine Kinase Inhibitors

Generally, RTKI resistance can be intrinsic (primary), when the tumor does not respond to treatment, or acquired (secondary), when resistance occurs after an initial response to RTKI treatment and a progressive selection of resistant tumor cells.

Most of the mechanisms of resistance to RTKIs are mainly attributed to mutations occurring within RTK itself. Several mutations were found in key residues (e.g., gatekeeper residue) in their catalytic domains, which prevents RTKI-binding to ATP-pocket of RTK by steric hindrance [14]. Clinical studies have revealed around fifty different mutations of BCR-ABL responsible for imatinib resistance, as well as the involvement of other elements of intracellular signaling; such as the forkhead box protein O1 (FOXO1), β-catenin, STAT3, the nuclear factor-kappa B (NF-κB), and AXL in RTKI resistance [45]. In gastrointestinal stromal tumors expressing KIT, genomic analysis of tumors have highlighted mutations in gatekeeper residues in BCR-ABL and EGFR, or activating mutations in BRAF, or insulin-like growth factor 1 receptor (IGF1R) amplifications were reported in imatinib resistance [46]. Some pre-clinical studies highlight other mechanisms, such as KIT mutations of the ATP-binding pocket of the kinase domain or the kinase activation loop [47,48].

Another way to circumvent the effect of RTKI is to activate a kinase located downstream or in parallel of the targeted signaling pathway, or on a parallel pathway. This latter is the most frequently described mechanism of resistance to RTKIs. This is particularly due to the emergence of compensatory signaling pathways when the major one is blocked, and to the existence of naturally occurring crosstalks and connections between different signaling pathways [49]. For instance, changes in signaling pathways are common with activation of EGFR or loss of PTEN (phosphatase and tensin homolog), but also with alternative activation of AKT/mTOR, of STAT3, and of vascular endothelial growth factor (VEGF) by RTKI-induced autocrine secretions of interleukins IL6 and IL8, and resistance [50]. KRAS mutation in NSCLC is a well-known mechanism of resistance to anti-EGFR RTKIs in clinic [51] and in pre-clinical studies. Thus, resistance of hepatocellular carcinoma (HCC) to sorafenib is linked with activation of EGFR. In addition, by promoting cell survival, Pi3K/AKT and autophagy are involved in therapeutic resistance of HCC. Furthermore, hypoxia induced by sorafenib treatment leads to the activation of extracellular signal-regulated kinases (ERK)/MAPK and JAK/STAT, and the up-regulation of hypoxy-inducible factor 2-alpha (HIF-2α), which in turn activates the transforming growth factor alpha (TGF-α) and EGFR [52]. Resistance of thyroid cancer to RTKIs involve RET mutations [53]. Frequent resistance mechanisms to BRAF inhibitors validated on patients’ tumors imply phosphorylation of EGFR, loss of PTEN, or activating mutations of MAPK, NRAS, or Pi3K-AKT [54].

Phenotypic transformation can lead to resistance to RTKIs. The most frequent example is the epithelial-mesenchymal transition (EMT) during which tumor cells lose their epithelial characteristics, such as cell-cell adhesion and polarity, in favor of mesenchymal characteristics and of the acquisition of an invasive phenotype. Thus, in NSCLC, translational studies have highlighted EMT as a mechanism of resistance to RTKIs of EGFR via the activation of EMT-specific signaling pathways (AXL and Hedgehog). Another rare transformation involved in the resistance of NSCLC to RTKIs is the histological transformation of a pulmonary adenocarcinoma into small cell lung cancer. These resistance mechanisms seem to be independent of the class of EGFR inhibitor used [23].

Another important mechanism of resistance is the selection of cancer cells expressing efflux pumps able to transport drugs. For example, in RCC, pre-clinical data showed that sorafenib and sunitinib can be sequestrated in the lysosome by ATP-binding cassette (ABC) transporter, P-glycoprotein [50]. Other mechanisms of resistance linked to the interaction of neoplastic cells with their microenvironment will be discussed in Section 3.3.

## 3. Improving the Use of Receptor Tyrosine Kinase Inhibitors: Combinatorial Treatment without Increasing Toxicity

As previously explained, the combination of RTKIs is ideal from a theoretical point of view, but is not easily achievable in clinical practice because of the toxicity. Therefore, combining a RTKI with another class of drug can be an interesting avenue. This section explains the rational bases for drug combinations in the light of pre-clinical analyzes of neoplastic cells and their microenvironment.

### 3.1. Receptor Tyrosine Kinase Inhibitor Combinations

In theory, combination of RTKIs is considered as an attractive option because it can prevent primary and secondary therapeutic resistance. The first strategy, called vertical pathway inhibition, aims to doubly inhibit the same signaling pathway. It consists in limiting the impact of a possible mutation of the targeted RTK or in activating downstream effectors. The choice of inhibiting the same target in two different ways was tested in a clinical trial with NSCLC patients treated with the first and third-generation anti-EGFR inhibitors. Currently, the only example of targeting an RTK and one of its downstream effectors is currently the double BRAF-MEK inhibition recommended in melanoma, but other combinations are still under investigation in clinical trial [27,55,56]. It should be noted that double inhibitions can be established on the basis of two selective RTKIs of a target or of a single RTKI with a double inhibitory action. For example, RO5126766, the first selective dual BRAF/CRAF and MEK inhibitor was investigated in a phase-1 dose-escalation clinical study [57].

A new strategy, called horizontal inhibition, is under development. It consists in a crosstalk inhibition to prevent the over-activation of a second pathway in response to the inhibition of the first one. Many pre-clinical data are encouraging and have given rise to phase-1 clinical trial in melanoma, combining an anti-BRAF or MEK RTKI with an anti-Pi3K. Other phase-1 agnostic clinical trials have investigated the combination of inhibitors of MEK and AKT. All these clinical trials illustrate very well the difficulty of combinations of RTKIs, since none has really resulted in a positive balance between increased toxicity and survival gain [57], as often seen in trials for combinations of RTKIs [15].

Toxicity of RTK inhibitors is therefore an integral part of the therapeutic challenge. Large part of their toxicity is attributable to their off-target effects. It should be prevented by a better determination of RTKI affinity for other targets, in order to select the most specific RTKIs [58]. Another approach consisting in reducing the distribution of RTKIs in healthy tissues using nanoparticles or pegylated liposomes is under intense research, while their clinical benefits remain to be proven [59].

### 3.2. Receptor Tyrosine Kinase Inhibitors and Synthetic Lethality

A combination of treatment leads to synthetic lethality when the single treatment did not cause cell death, unlike their combination. One of the first successes in synthetic lethality was the poly-adenosine diphosphate ribose polymerase (PARP) inhibitor, which is currently used in clinic to achieve synthetic lethality in breast cancer with mutated (BRCA)1 or 2. Recently, Maifrede et al. have reported that in acute myeloid leukemia, inhibition of Fms-like tyrosine kinase 3 (FLT3) by an RTKI appears to downregulate key proteins in DNA double-strand break (DSB) repair; such as BRCA1, BRCA2; and RAD51. In this study, combination of RTKI of FLT3 with a PARP inhibitor has shown very encouraging results in mouse models [60].

This concept can be extended to other therapeutic classes such as metabolic inhibitors. Ding et al. thus identified by clustered regularly interspaced short palindromic repeats (CRISPR) knock-out screening that transaldolase, an enzyme of the non-oxidative pentose phosphate pathway, was essential for the survival of tumor cells treated with lapatinib. Inactivation of this enzyme combined with inhibition of HER2 reduced the level of nicotinamide adenine dinucleotide phosphate (NADPH), and thus increased the production of reactive oxygen species (ROS) while reducing the synthesis of lipids and nucleotides [61].

The regulatory pathways of apoptosis could also be involved in drug resistance. Arsenic trioxide (ATO) is successfully used in a rare form of leukemia, acute promyelocytic leukemia, as it induces the differentiation of tumor cells. Wang et al. have noticed that ATO produces numerous apoptotic signals without succeeding in the induction of apoptosis, because it causes phosphorylation and therefore inactivation of glycogen synthase kinase (GSK)3β, one of the key pro-apoptotic enzymes. Then, they successfully tested sorafenib, which activated GSK3β, increased the rate of apoptosis, and significantly prolonged the survival rate of mouse tumor models [62].

### 3.3. Impact of the Non-Immune Microenvironment on Receptor Tyrosine Kinase Inhibitor Efficacy

RTKIs targeting tumor cells also act on cellular components of TME. This latter is indeed also influenced by chemotherapy, immunotherapy, and radiotherapy, and in turn influences the response to treatment [63].

Stromal cells in TME exposed to RTKIs produce cytokines, hormones, or growth factors that modulate the response of the tumor to RTKIs. Thus, RTKIs targeting focal adhesion kinase (FAK), FGFR, c-MET, and VEGFR decrease the number of fibroblasts or their activation, and therefore their role in supporting growth of various tumors [64,65].

Interestingly, the TME can itself modify the signaling of neoplastic cells and drive resistance to RTKIs. In thyroid carcinoma, pericytes lead to vemurafenib resistance through secretion of thrombospondine 1 (TSP-1) and transforming growth factor beta-1 (TGFβ1), which increase expression of protein kinase R (PKR)-like endoplasmic reticulum kinase (pERK1/2), phosphorylated AKT (pAKT), and phosphorylated mothers against decapentaplegic homolog 3 (pSMAD3) levels [66]. Similarly, stromal cells can secrete hepatocyte growth factor (HGF) which activates MET that in turn stimulates MAPK and Pi3K/AKT/mTOR, leading to BRAFi resistance of melanoma cells [67,68]. Resistance to RTKI therapy targeting HER2 could be mediated by cancer-associated fibroblasts (CAFs) through the secretion of neuregulin-1 beta (NRG1β), which is an HER3 ligand [69].

PDGFR and VEGFR inhibitors administered in small doses induce vascular normalization which improves drug distribution within the tumor. The depletion of pericytes and hypoxia caused by sunitinib treatment may increase the metastatic spread of tumor cells [64].

Few studies have looked at the effects of RTKIs on the extracellular matrix (ECM), but there is an inverse correlation between the efficacy of lapatinib in HER2+ breast cancer and the elastic modulus of ECM, which means that the more effective lapatinib, the more easily the ECM deforms [70].

### 3.4. Impact of the Immune Microenvironment on Receptor Tyrosine Kinase Inhibitor Efficacy

Immune microenvironment is also modulated by RTKIs. For example, dasatinib, sorafenib, and imatinib decrease T-regulator cells (Tregs) and increase anti-tumor T-cell response. Similarly, sunitinib decreases the survival and expansion of myeloid-derived suppressor cells (MDSCs) and M2 macrophages, promoting the establishment of a permissive immune-competent TME [64,71]. Cabozantinib stimulates neutrophil-mediated anticancer innate immune response [72]. Treatment with BRAF inhibitors alone or with MEK inhibitors increases the level of tumor associated CD8 + lymphocytes and melanoma antigens expression [73]. For example, RTKIs targeting FGFR, in addition to their action on neoplastic cells, reduce the number of immunosuppressive MDSCs in the tumor and induce senescence of cancer-associated fibroblasts (CAFs) [65]. VEGFR1 inhibitors, in addition to their anti-angiogenic actions, can normalize tumor microvasculature and decrease the infiltration of MDSCS, Treg lymphocytes, and some populations of immunosuppressive tumor-associated macrophages (TAMs) [71,74].

Furthermore, establishment of an immunosuppressive TME was observed during the acquisition of resistance to RTKIs. Thus, resistance of BRAF tumors to RTKIs is concomitant with an increase in MDSCs. Additionally, the resistance of glioblastoma to axitinib led to an increased number of Treg lymphocytes and the expression of the programmed cell death protein-1 (PD-1) inhibitory checkpoint. A shift in TAMs towards a phenotype promoting tumor growth was observed in gastro-intestinal stromal tumors (GISTs) becoming resistant to imatinib [64]. In addition, the RTKIs targeting EGFRs seem less effective in patients with a tumor highly infiltrated by CD8+ lymphocytes and associated with a high level of programmed death-ligand 1 (PD-L1) [75]. Taken together, these results indicate that an anti-tumor activity of immune TME seems important in the effectiveness of RTKIs [75]. In addition, the immune cells of the microenvironment can act through other mechanisms than immunity. Thus, MDSCs recruited under treatment can produce pro-angiogenic factors and stimulate VEGF-independent angiogenesis [76].

There is therefore a rational for developing combinations with RTKIs and immunotherapies. Few clinical studies have already been published on the subject and the toxicity of these combinations often seems to be very high. However, a phase-3 trial combining atezolizumab (anti-PD-L1) with sunitinib (VEGFR inhibitor) in metastatic RCC, and a phase-2 trial combining pembrolizumab (anti-PD1) with dabrafenib (BRAF inhibitor) and trametinib (MEK inhibitor) in BRAF-mutant melanoma patients have shown encouraging results with moderate toxicity [64]. Similar results were obtained with the combination of lenvatinib and pembrolizumab in a phase-2 clinical study in endometrial and kidney cancer [77], as well as with the combination of nivolumab and regorafenib, evaluated in a phase-2 clinical study in advanced gastric or colorectal cancers [78].

Tyrosine kinase receptors expressed by non-neoplastic cells may also become an attractive therapeutic target, such as AXL, the RTK expressed by TAMs, which is considered as an emerging class of innate immune checkpoints [79]. In view of all these elements, some early clinical studies are currently studying some RTKI and checkpoint inhibitor combinations (e.g., nivolumab combined with pazopanib or sunitinib, axitinib combined with pembrolizumab) [80,81,82]. The relationships between RTKIs and immune cells, as well as clinical trials combining RTKIs with immune checkpoints inhibitors are summarized in Table 2.

## 4. Conclusions

**Figure 3:**
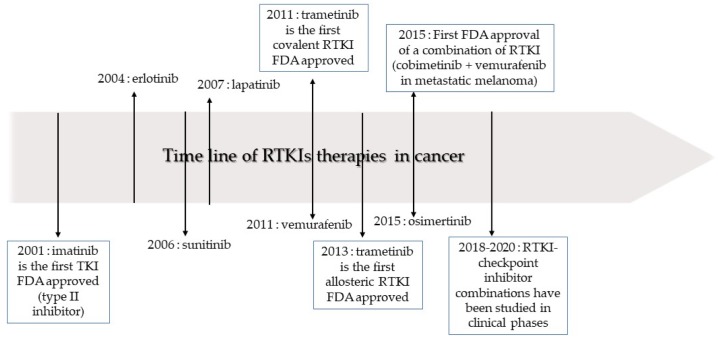
Time line of receptor tyrosine kinase inhibitor (RTKI) development and approval for the treatment of cancer.

RTKIs have revolutionized the practice of oncology and hematology other the past 20 years with over 40 compounds approved by the FDA (Figure 3). However, apart from rare exceptions, such as some cases of chronic myeloid leukemia, no patient can currently be cured by the use of RTKI as single agent in therapy. The problems of the emergence of resistance to treatment and toxicity, leading to the reduction of the given dose or to RTKI treatment discontinuation, are the main challenges for their use in cancer patients. With the current growth in the cost of treatment that discourages access to care, reduction of development costs should also be considered as a priority.

Production of new RTKIs with different mechanisms of action, such as covalent inhibitors, inhibitors resistant to the most frequent tumor mutations, or inhibitors inducing RTK degradation or internalization, is a promising approach. Reducing side effects due to “off-target” effects by improving the selectivity of RTKIs is another major aspect to consider. The time for the new RTKI development, and therefore their cost, may be reduced by the use of artificial intelligence (AI). Indeed, machine learning also offers new possibilities to predict the 3D structure of a protein from the sequence of its amino acids, interactions and binding between molecules of interest, and finally to design new potential drugs [83,84]. A first example of discovery of RTKI by artificial intelligence was given by Zhavoronkov et al. in 2019. A system of deep learning made it possible to discover several candidate inhibitors of the discoidin domain receptor 1 in 21 days. Among them, two were effective in vitro and one showed interesting results in mouse models [85]. After a design of the new molecules assisted by AI, their initial development can be made more efficient by new microfluidic techniques. Desai et al, for example, produced new ABL inhibitors. Compounds selected as the most promising by algorithms are synthesized automatically in microarrays or microfluidic platforms and then screened by the determination of the IC50 and other parameters. The best candidates can then directly be integrated in pre-clinical studies [86,87,88].

Research for an effective and low-toxic combination is very complex. So far, apart from the BRAF-MEK combination in melanoma, few combinations of two RTKIs have been used clinically. Production of more selective RTKIs may increase the tolerance of their combination.

Another promising approach is the combination of RTKIs with another class of inhibitors. Analysis of such combinations must take into account the effects of synthetic lethality type and the effects on TME. This involves analysis of large databases, which can be performed by various AI techniques, and new in vitro models as organs-on-chips. These systems include microchannels continuously perfused by a culture medium and containing different cell types organized in organ-specific, tissue-tissue interfaces [89]. Large screening of additives or synergistic properties of an RTKI with another or with other drugs, taking into account the effect of RTKIs on the immune and nonimmune microenvironment will likely help in the development of effective therapeutics with low toxicity and cost for a better care of cancer patients. 

## Figures and Tables

**Table 1:** List of FDA-approved small molecule protein kinase inhibitors (updated by 18 August 2019). These inhibitors are sorted by their main targeted pathways and from signal initiation at cytoplasmic membrane of the cell to its propagation to the nucleus [13,15,16,17,18,19].

Name	Known Target	Inhibitor Class	Indications
**Anaplastic lymphoma kinase (ALK)**			
Alectinib	ALK and RET	II	ALK+ NSCLC
Brigatinib	ALK, ROS1, IGF-1R, Flt3, EGFR	I	ALK+ NSCLC after crizotinib
Ceritinib	ALK, IGF-1R, InsR, ROS1	II	ALK+ NSCLC as first-line treatment or after crizotinib resistance
Crizotinib	ALK, c-Met (HGFR), ROS1, MST1R	II	ALK+, ROS1+ NSCLC
Entrectinib	TRKA/B/C, ROS1, ALK	I	ROS1+ NSCLC; solid tumors with NTRK fusion proteins
Lorlatinib	ALK	I	ALK+ NSCLC
**Fusion of breakpoint cluster region and Abelson (BCR-ABL)**			
Bosutinib	BCR-ABL, Src, Lyn, Hck		CML
Dasatinib	BCR-ABL, EGFR, Src, Lck, Yes, Fyn, Kit, EphA2, PDGFRβ	I	Ph+ chronic ML and ALL
Imatinib	BCR-ABL, Kit, PDGFR	II	Ph+ CML or ALL, CEL, DFSP, HES, GIST, MDS/MDP
Nilotinib	BCR-ABL, PDGFR, DDR1	II	Ph+ CLL
Ponatinib	BCR-ABL, BCR-ABL T315I, VEGFR, PDGFR, FGFR, EphR, Src family kinases, Kit, RET, Tie2, Flt3	II	Ph+ CML or ALL
**Epidermal growth factor receptor (EGFR)**			
Afatinib	EGFR, ErbB2, ErbB4	Covalent (V)	NSCLC
Dacomitinib	EGFR/ErbB2/ErbB4	I	EGFR- mutated NSCLC
Erlotinib	EGFR	I	SCLC and PaC
Gefitinib	EGFR	I	NCLC
Lapatinib	EGFR, ErbB2	II	BC
Neratinib	ErbB2/HER2	Covalent (V)	HER2+ breast cancer
Osimertinib	EGFR T970M	Covalent (V)	NSCLC
Vandetanib	EGFRs, VEGFRs, RET, Brk, Tie2, EphRs, Src family kinases	I	MTC
**FMS-like tyrosine kinase 3 (FLT3)**			
Gilteritinib	FLT3	I	AMLwith FLT3 mutation5
Midostaurin	FLT3	I	ALL Flt3 mutation+
Fibroblast growth factor receptors (FGFR)			
Erdafitinib	FGFR1/2/3/4	I	Urothelial carcinoma
Janus kinase (JAK)			
Ruxolitinib	JAK1 and 2	I	MF and PV
**Neurotrophic Tyrosine Receptor Kinase (NTRK)**			
Larotrectinib	NTRK	I	Solid tumors with NTRK gene fusion proteins
**Vascular endothelial growth factor (VEGFR)**			
Axitinib	VEGFR1/2/3, PDGFRβ	II	RCC
Carbozantinib	RET, Met, VEGFR1/2/3, Kit, TrkB, Flt3, Axl, Tie2, ROS1	I	Metastatic MTC, advanced RCC and HCC
Lenvatinib	VEGFRs, FGFRs, PDGFR, Kit, RET	II	DTC
Pazopanib	VEGFR1/2/3, PDGFRα/β, FGFR1/3, Kit, Lck, Fms, Itk	I	RCC, STS
Regorafenib	VEGFR1/2/3, BCR-ABL, BRAF, BRAF(V600E), Kit, PDGFRα/β, RET, FGFR1/2, Tie2, Eph2A	II	CRC, GIST
Sorafenib	B/C-Raf, BRAF (V600E), Kit, Flt3, RET, VEGFR1/2/3, PDGFRβ	II	RCC, DTC and HCC
Sunitinib	PDGFRα/β, VEGFR1/2/3, Kit, Flt3, CSF-1R, RET	II	RCC, GIST, PNET
**BRAF**			
Dabrafenib	BRAF	I	Melanoma and NSCLC with BRAF mutations
Encorafenib	BRAFV600E/K	I	BRAFV600E/K mutant melanoma with binimetinib
Vemurafenib	A/B/C-Raf, BRAF (V600E), SRMS, ACK1, MAP4K5, FGR	I	Melanoma with BRAFV600E mutation and ECD
**Bruton tyrosine kinase**			
Acalabrutinib	Bruton tyrosine kinase	Covalent (V)	MCL
Ibrutinib	Bruton tyrosine kinase	Covalent (V)	MCL, CLL, WM, graph vs host disease.
**Mitogen-activated protein kinase kinase (MEK)**			
Binimetinib	MEK1/2	III	BRAF V600E/K melanoma with encorafenib
Cobimetinib	MEK1/2	III	Melanoma with BRAF V600E/K mutations with vemurafenib
Trametinib	MEK1/2	III	Melanoma (2013) and NSCLC (2017) with BRAF mutations
**Cyclin-dependent-kinase 4/6**			
Abemaciclib	CDK4/6	I	HR+, HER– BC
Palbociclib	CDK4/6	I	ER+ and HER2– BC
Ribociclib	CDK4/6	I	HR+-EGFR– metastatic BC

Type-I inhibitors are ATP-competitive inhibitors that bind to active conformations, Type-II inhibitors bind to a site adjacent to the ATP site of inactive kinases and maintain their inactive conformation; Type-III inhibitors are allosteric inhibitors, they are highly selective and inhibit kinases by binding to an allosteric site, distant from the ATP site and the hinge; Type-IV inhibitors target substrate-binding site in a reversible manner (under development); Type-V inhibitors bind to their targets with covalent bonds. ALL: acute lymphoid leukemia; ALK: Anaplastic lymphoma kinase; BCR-ABL: breakpoint cluster region- Abelson; CDK: cyclin-dependent kinase; CML: chronic myeloïd leukemia; CLL: chronic lymphoid leukemia; CRC: colorectal carcinoma; CSFR: colony stimulating factor 1 receptor; DTC: differentiated thyroid carcinoma; ECD: Erdheim-Chester disease; EGFR: Epidermal growth factor receptor; EphR: Ephrin receptor; FGFR: fibroblast growth factor receptor; FKBP: FK506-binding protein; GIST: gastro intestinal stromal tumor; HCC: hepatocellular carcinoma; HER or ErbB: human epidermal growth factor receptor; HGFR or c-Met: hepatocyte growth factor receptor; InsR: Insulin receptor precursor; IGF insulin-like growth factor; Itk: interleukine 2 inducible T cell kinase; JAK: Janus kinase; MAPK: mitogen-activated protein kinases; MCL: mantle cell lymphoma; MEK: MAPK/extracellular signal-regulated kinase; MF: Myelofibrosis; ML: myeloid leukemia; MTC: medullary thyroid cancer; mTOR: mechanistic target of rapamycin; NSCLC: non-small-cell lung cancer; NTRK: neurotrophic tyrosine receptor kinase; PDGFR: Platelet-derived growth factor receptors; PNET: primitive neuroectodermal tumor; RCC: renal cell carcinoma; SGCAs: subependymal giant cell astrocytoma; SCLC: small cells lung carcinoma; SRMS: src-related kinase lacking C-terminal regulatory tyrosine and N-terminal myristylation sites; STS: soft tissue sarcoma; Tie2: tyrosine kinase with immunoglobulin and EGF homology domains; TRK: tropomyosin receptor kinase; TSGCTs: tenosynovial giant cell tumors VEGFR; WM: Waldenstrom’s macroglobulinemia.

**Table 2:** Relationship between anti-tumor immunity and RTKIs [64,65,71,73,74,76,77,78,79,80,81,82].

**RTKI actions in favor of an anti-tumor immune response**		
**RTKIs**	**Effects on immune cells**	**Characteristics of carried-out studies**
BRAF inhibitors +/- MEK inhibitors	↑ CD8+ TIL and melanoma antigen expression	Patient biopsies and in vivo pre-clinical study, BRAF mutated melanoma
Cabozantinib	↑ neutrophil-mediated antitumor innate immunity	In vivo pre-clinical study, murine prostate cancer
Dasatinib	↓ MDSCs	Patient biopsies and in vivo pre-clinical study, CML
Sorafenib	↓ MDSCs	Patient biopsies and in vivo pre-clinical study, HCC
FGFR inhibitors	↓ MDSCs	In vivo pre-clinical study, murine breast cancer
Sunitinib	↓ MDSCs and M2 macrophages	In vivo pre-clinical study, RCC
VEGFR1 inhibitors	↓ MDSCs, Tregs and M2 macrophages	In vivo pre-clinical studies on RCC and NSCLC
**Tumor immune tolerance observed during the acquisition of resistance to RTKI**		
**RTKI**	**Effects on immune cells**	**Characteristics of studies carried out**
Axitinib	↑ Tregs, ↑ and PD-1 expression	In vivo pre-clinical study, glioblastoma
BRAF inhibitors	↑ MDSCs	In vivo pre-clinical study, BRAF mutated melanoma
Imatinib	↑ M2 macrophages	In vivo pre-clinical study, GIST
**Combinations of RTKI and checkpoint inhibitors under investigation**		
**RTKI**	**Checkpoint inhibitors**	**Clinical trial**
Dabrafenib	Pembrolizumab (anti-PD1)	Phase-2 trial, B-ref mutated melanoma
Lenvatinib	Pembrolizumab (anti-PD1)	Phase-2 trial, endometrial cancer and RCC
Regorafenib	Nivolumab	Phase-2 trial, gastric or colorectal cancer
Sunitinib	Atezolizumab (anti-PD-L1)	Phase-3 trial, metastatic RCC

CML: chronic myeloid leukemia; GIST: gastro intestinal stromal tumor; HCC: hepatocellular carcinoma; MDSCs: myeloid-derived suppressor cell; MEK: mitogen-activated protein kinase; NSCLC: non-small-cell lung cancer; PD1: progammed cell death protein 1; PD-L1: programmed death-ligand 1; RCC: renal cell carcinoma; TILs: tumor-infiltrating lymphocytes; Tregs: regulatory T cells.
